# Department of Practice of Medicine

**Published:** 1895-12

**Authors:** 

**Affiliations:** Galveston


### Current Medical Literature.

### DEPARTMENT OF PRACTICE OF MEDICINE.

EDITED BY PROF. ALLEN J. SMITH, M. D., GALVESTON.

FISTULA IN ANO AS A COMPLICATION OF PULMONARY TUBERCULOSIS. —Wells (*Charlotte Medical Journal*, October) comes to the following conclusions in a paper upon the above subject:

1. That *fistula in ano* occurs oftener in the subjects of pulmonary tuberculosis than in the case of the otherwise healthy or those affected with other diseases.

2. That the fistula in these cases may be either tubercular or non tubercular in its nature. If the former it is difficult to cure by an operation, and if an operation is decided upon, it should be more radical than those heretofore in vogue,—experience and authority have condemned the ordinary operation of simple incision in these cases. If the fistula is free from local tubercular involvement, an operation is advisable and it will be generally successful.

3. That *fistula in ano* is in no sense a salutary issue, and that every operable case, which is annoying to the patient, should be cured, if possible, and that endeavors should be made to so improve operative measures as to permit the subjects of tubercular fistula to be operated upon with a fair promise of success.

DISAPPEARANCE OF THE FIRST SOUND OF THE HEART IN TYPHOID FEVER.—Mongour, of Bordeaux, (*Archives Clinique de Bordeaux*, September, 1895) in an article based upon two cases of typhoid fever (in one of which diphtheria was a complication) in which the first cardiac sound was lost, after commenting upon these two cases and reviewing the physiology of the heart sounds and the scant literature upon the subject, as well as analyzing the features of a number of cases of typhoid fever and other diseases in which the absence of the first cardiac sound sometimes occurs, both in relation to the symptomatology and post mortem appearances, comes to the following conclusions:

1. The disappearance of the first sound of the heart in typhoid fever seems to be due to the existence of a myocarditis.

2. If this disappearance coincides with an acceleration of the cardiac rhythm, one may infer, besides the myocarditis, the action of microbic poisons upon the accellerator or inhibitive nervous centres or upon the peripheral vessels. This special determination of toxic action would seem more grave than the myocarditis, which, in most cases, is apt to recover entirely. The disappearance of the first sound, either at the apex or at the base, at any time in the course of typhoid fever, has no grave prognostic significance, if the pulse does not exceed 110 as a maximum; if the tachycardia exceeds this limit the disappearance of the systolic sound may be considered a fatal symptom.

---

UNIQUE FORM OF MOTOR PARALYSIS DUE TO COLD.—(*Med. News*, August 25, 1894.) Dr. E. C. Rich describes a peculiarity running through five generations of his own family, consisting of a tonic spasm of certain muscles, or set of muscles, provoked by exposure to cold. The condition seems to be more readily induced in one subject it when the general system is reduced from fatigue, previous or present illness, or insufficient food. At date of author's article there were fourteen members of this family living and occupied in farming and stock-raising in the southern part of Idaho, and records of eight more, now dead, who had inherited the affection. The individual affected is represented as in perfect health during the intervals between the attacks, which comes on not as in Thomson's disease, during voluntary motion of the muscles involved, but, during repose. As long as the muscles are kept in motion the stiffness does not occur; but, for example, upon setting down after a walk in the cold the leg muscles may become stiff and helpless although the thigh muscles may remain normal. The hand or arm, or perhaps, but a single finger, may be involved, but occasionally the entire body, the face not escaping, may be rendered helpless for a time by the disorder. Upon placing snow in the mouth the tongue is apt to become tonically convulsed, but the warmth of the cavity restores it in a few minutes. The application of heat to a spasmodic part always promptly relieves the condition. The affection is not accompanied by any signs of inflammation; but there is usually some slight swelling of the sitffened member, probably from vaso-motor disturbance. The trouble has not been known to skip a generation and appear in the next, the inheritance being direct or failing altogether.

---

GOUT AND THE TEETH.—(*Philadelphia Times and Register*, May 19, 1894.) Henry Burchard, a dental surgeon, in summing up a paper upon the relations of gout and erosion of the teeth and *pyorrhœa alveolaris* makes the following statements: There is an affection of the the teeth for which explanation as to local causation are insufficient. This has been noted by both dentists and physicians to be associated with gout, there being in many instances history of heredity and acute outbreaks, and some cases show decided evidence of lithæmia. After the removal of all visible sources of irritation, the dental disease persists or recurs after a slight lessening of severity of the local symptoms. The teeth of certain individuals with or without a definite history of gout become susceptible to periodical inflammation, and and this in the absence of local causes for irritation. The ingestion of undue amounts of nitrogenous foods or heavy wines may be followed by one of these attacks of pericementitis, and the removal of these articles from the dietary be followed by amelioration. It seems probable that this local inflammation is due to the formation and retention in the cementum, the periosteum of the tooth, of some substances which should be eliminated, just as it takes place in the joints in gout. In about seventy-five per cent of these cases of erosion or pharadennic pericementitis dentists give an unfavorable prognosis, and in spite of all local measures the results justify such a belief. The treatment recommended involves the removal of any dead or foreign matter about or in the teeth, the destruction of all bacteria, the splinting of loose teeth to fix and rest these organs, the correction of altered secretion of the glands of the parts about the teeth, the correction of gastric and intestinal catarrh, and the administration of antilithæmic remedies, especially the tartrate of lithium, which acts as an antilithæmic and also as a mild laxative.

